# Takotsubo syndrome following mitral valve surgery in a paediatric patient: a case report

**DOI:** 10.1093/ehjcr/ytaf167

**Published:** 2025-04-07

**Authors:** María Victoria Piovano, Juan Cruz Ponceliz, Benjamin Chiostri, Guillermo Nuncio Vaccarino, Christian Kreutzer

**Affiliations:** Department of Cardiology, Hospital Universitario Austral, Av. Perón 1500, Pilar, Buenos Aires 1629, Argentina; Department of Cardiovascular Surgery, Hospital Universitario Austral, Av. Perón 1500, Pilar, Buenos Aires 1629, Argentina; Pediatric Cardiovascular Surgery, Hospital Universitario Austral , Av. Perón 1500, Pilar, Buenos Aires 1629, Argentina; Institute of Cardiology and Cardiovascular Therapy, Hospital Universitario Austral, Av. Perón 1500, Pilar, Buenos Aires 1629, Argentina; Pediatric Cardiovascular Surgery, Hospital Universitario Austral , Av. Perón 1500, Pilar, Buenos Aires 1629, Argentina

**Keywords:** Takotsubo syndrome, Mitral valve surgery, Case report, Cardiogenic shock

## Abstract

**Background:**

Takotsubo syndrome is an uncommon condition in children, mainly triggered by physical stressors. However, it has never been described after a mitral valve surgery in this population.

**Case summary:**

We present the case of a 14-year-old boy with a history of Loeys–Dietz syndrome, who had previously undergone aortic root replacement due to an aortic aneurysm. He was admitted for elective mitral valve repair due to asymptomatic severe primary mitral regurgitation caused by bileaflet prolapse. Mitral valve repair was performed without incidences, but in the postoperative, he developed cardiogenic shock and cardiorespiratory arrest. Echocardiography showed severe left ventricular dysfunction with mid to distal diffuse hypokinesia, with better contractility in basal segments. Extracorporeal membrane oxygenation was required for 48 h, after which his haemodynamic status improved, leading to a gradual reduction in supportive measures. Control echocardiogram showed partial recovery of the left ventricular function with improved contractility in apical segments. The patient could be discharged at 2 weeks. One month later, he remained asymptomatic, with a normal left ventricular ejection fraction.

**Discussion:**

Takotsubo syndrome can be a life-threatening condition, with a higher prevalence of acute complications in male patients. Reports of this syndrome after cardiac surgery have mostly involved the mitral valve, which could be explained by the abrupt change in ventricular loading conditions and increase in afterload. The immediate postoperative use of epinephrine and dobutamine has been identified as a risk factor for the development of Takotsubo syndrome. Despite initially considered a benign entity, Takotsubo syndrome can be a life-threatening condition, with a mortality risk similar to patients presenting with acute coronary syndromes. Mitral valve surgery may serve as a trigger for Takotsubo syndrome and should be considered as a potential differential diagnosis in patients presenting with postoperative cardiogenic shock.

**Learning points**

- Despite initially considered a benign entity, Takotsubo syndrome can be a life-threatening condition, with a mortality risk similar to patients presenting with acute coronary syndromes.
- Mitral valve surgery may serve as a trigger for Takotsubo syndrome and should be considered as a potential differential diagnosis in patients presenting with postoperative cardiogenic shock.

## Introduction

Takotsubo syndrome, initially described in 1990, is characterized by a temporary wall motion abnormality of the left ventricle that extends beyond a single epicardial vascular distribution and can be triggered by emotional or physical stressors. Several anatomical variants have been described, with the apical ballooning type being the most common. It is more frequently seen in post-menopausal women, with few cases reported in children.^1^

Although the precise pathophysiological mechanisms of this syndrome remain incompletely understood, substantial evidence suggests that sympathetic stimulation is central to its pathogenesis.^2^

## Summary figure

**Table:** 

Day 1 Morning	Admission for elective cardiac surgery due to asymptomatic severe primary mitral regurgitation. Previous left ventricular ejection fraction (LVEF) was 60% with moderate dilatation of the left ventricle (LV). Previous computed tomography (CT) angiography showed no coronary lesions. Mitral valve repair was performed without incidences. Norepinephrine (0.3 μg/kg/min) and epinephrine (0.1 μg/kg/min) were required after extracorporeal circulation.
Day 1 Evening	The patient presented with cardiogenic shock. Milrinone was initiated, resulting in initial improvement.
Day 3	The patient experienced a cardiorespiratory arrest, after which his haemodynamic status severely deteriorated. Electrocardiogram showed inverted T waves in precordial leads. An echocardiogram revealed severe LV dysfunction (LVEF 30%) with mid to distal diffuse hypokinesia. Extracorporeal membrane oxygenation (ECMO) was required.
Days 4–5	Gradual improvement in haemodynamic status led to discontinuation of ECMO
Day 7	The patient was extubated. Serial echocardiographic evaluations demonstrated ongoing improvement in basal segments.
Day 10	Echocardiography showed partial recovery of LV systolic function, with an LVEF of 45%–50% and improved contractility in apical segments.
Day 14	Discharge.
Day 30	The patient remained asymptomatic, with a normal LVEF (59%).

## Case presentation

We present the case of a 14-year-old boy with a history of Loeys–Dietz syndrome, who had undergone valve-sparing aortic root replacement 2 years earlier due to an aortic aneurysm. He was admitted for elective mitral valve repair due to asymptomatic severe primary mitral regurgitation caused by bileaflet prolapse, with the posterior leaflet predominantly affected. Although mitral valve prolapse was present at the time of the previous surgery, no regurgitation was evident. Due to the patient’s irregular follow-up, it is unclear when the regurgitation began.

An echocardiogram performed a month prior showed a left ventricular (LV) ejection fraction (LVEF) of 60%, moderate dilatation of the LV (diastolic diameter 65 mm and systolic diameter 43.5 mm), and a myxomatous mitral valve with annular dilation and bileaflet prolapse, resulting in severe mitral regurgitation. Computed tomography (CT) angiography prior to surgery revealed no coronary lesions.

Mitral valve repair involved re-sternotomy, triangular resection of the posterior leaflet, and prosthetic ring annuloplasty, with a cardiopulmonary bypass time of 74 min and a cross-clamp time of 59 min. Myocardial protection was established through the administration of anterograde Del Nido cardioplegia. Left ventricular vent was placed via the right superior pulmonary vein. Intraoperative transoesophageal echocardiography confirmed normal LVEF and functioning of the repaired valve, and extracorporeal circulation was discontinued (Supplementary material online).

Norepinephrine (0.3 μg/kg/min) and epinephrine (0.1 μg/kg/min) were required post-extracorporeal circulation. In our centre, epinephrine is routinely administered following surgery with extracorporeal circulation. In this case, norepinephrine was added due to vasoplegia. Upon arrival at the intensive care unit, the patient presented with cardiogenic shock: cold extremities, oliguria, tachycardia, metabolic acidosis, and elevated serum lactate. Milrinone was initiated, resulting in initial improvement; however, high doses of norepinephrine were still required. Lactic acid levels decreased from 15.9 mmol/L at 8 a.m. to 2.5 mmol/L at 8 p.m. (normal range: 0.5–2.2 mmol/L). Although his condition seemed to be improving, he suddenly experienced a cardiorespiratory arrest 48 h post-surgery, after which his haemodynamic status severely deteriorated. Cardiopulmonary resuscitation manoeuvres were performed for 5 min, resulting in a return of spontaneous circulation; nevertheless, the patient required high doses of inotropes and vasopressors (epinephrine 0.8 mcg/kg/min and norepinephrine 1 mcg/kg/min).

Electrocardiogram showed inverted T waves in precordial leads. An echocardiogram revealed severe LV dysfunction (LVEF 30%) with mid to distal diffuse hypokinesia, showing better contractility in basal segments. Extracorporeal membrane oxygenation (ECMO) was established by peripheral cannulation in the Intensive Care Unit with an initial flow rate of 50 mL/kg/min. No LV venting strategies were established. Daily echocardiographic assessments showed consistent findings resembling the initial evaluation. After ECMO was initiated, haemodynamic status improved, leading to a gradual reduction in supportive measures. Vasopressors were promptly de-escalated to adrenaline 0.2 mcg/kg/min and noradrenaline 0.2 mcg/kg/min. Six hours later, noradrenaline was reduced to 0.1 mcg/kg/min. These doses remained stable for 30 h, after which adrenaline could be further reduced to 0.1 mcg/kg/min. Flow rate was gradually reduced and ECMO was finally discontinued after 48 h. The patient was extubated on the seventh postoperative day.

Serial echocardiographic evaluations demonstrated ongoing improvement in basal segments, although LVEF remained severely depressed until the 10th postoperative day (Supplementary material online). Echocardiography on Day 10 showed partial recovery of LV systolic function, with an LVEF of 45%–50% and improved contractility in apical segments. Two weeks later, the patient was discharged. One month later, he remained asymptomatic, with a normal LVEF (59%).

## Discussion

Although initially considered a benign entity due to its reversible nature, the mortality risk of Takotsubo syndrome is similar to that of patients presenting with acute coronary syndromes.^1^ The prevalence of acute complications such as cardiogenic shock and cardiac arrest is higher in male patients, who coincidentally have a higher mortality.^3^

Regarding diagnostic criteria, there have been variations over the years. The Heart Failure Association criteria, International Takotsubo Diagnostic Criteria (InterTAK criteria), and Mayo Clinic criteria coincide in the presence of transient wall motion abnormalities extending beyond a single epicardial vascular distribution and the necessity of excluding myocarditis. However, the InterTAK Diagnostic Criteria introduced some changes:

1. An emotional, physical, or combined trigger is not obligatory.
2. Neurologic disorders (e.g. subarachnoid haemorrhage, stroke/transient ischaemic attack, or seizures) and pheochromocytoma may serve as triggers for Takotsubo syndrome.
3. Rare cases exist without any electrocardiographic changes.
4. Significant coronary artery disease is not a contraindication in Takotsubo syndrome.^2^

Differential diagnosis such as poor myocardial protection and coronary air embolism should be considered in this patient, although unlikely considering normal LV function after weaning off cardiopulmonary bypass and the lack of correlation with a coronary artery territory. Angiography was not performed due to haemodynamic instability and the improbability of the diagnosis, given the absence of anatomic correlation. Additionally, the patient had undergone a CT angiography prior to surgery, which revealed no coronary lesions or anomalies.

Reports of Takotsubo syndrome after cardiac surgery have mostly involved the mitral valve. A previous review identified that most of the patients were postmenopausal women and that the presentation was usually early after surgery and often dramatic.^4^ A case-control study identified that the immediate postoperative use of epinephrine and dobutamine and atrioventricular valve surgery were factors associated with the development of Takotsubo syndrome.^5^

A systematic review proposed two main mechanisms to explain the association between Takotsubo syndrome and mitral valve surgery:

1. The abrupt change in ventricular loading conditions when chronic atrioventricular regurgitation is suddenly corrected may lead to a sharp increase in endogenous catecholamines.
2. The sudden decrease in ventricular preload and increase in afterload leads to a mismatch in myocardial mechanics, a peak in ventricular myocardial stress, and a transient increase in myocardial oxygen consumption.^6^

Concerning the prevalence of Takotsubo syndrome in children, it is an uncommon condition, occurring mainly in adolescents with underlying psychiatric disorders. It generally presents with physical stressors as triggers, such as pregnancy, medications (catecholamines/anaesthetics), or drugs of abuse.^2^ Currently, there are no specific guidelines regarding the diagnosis and management of Takotsubo cardiomyopathy in children, but the presentation, electrocardiogram, and imaging findings are comparable with those in adults.^7^

The prognosis of Takotsubo cardiomyopathy depends on the tolerance and management of the acute phase. Management of patients with cardiogenic shock depends on the presence of LV outflow tract obstruction (LVOTO). When LVOTO is present, inotropes must be avoided, and ECMO should be considered for refractory cases.^1^

## Conclusion

Despite advances in the understanding of Takotsubo syndrome, the exact pathophysiological mechanisms remain unclear. Mitral valve surgery has lately been identified as a potential trigger, with most cases occurring in postmenopausal women. Although rare, we should consider this syndrome as a differential diagnosis in patients with postoperative cardiogenic shock.

## Lead author biography


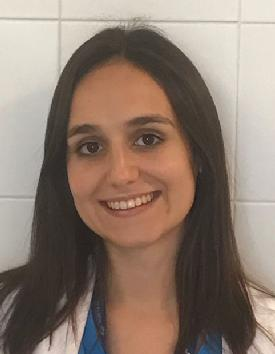


María Victoria Piovano is currently working in Hospital Universitario Austral as a cardiology resident. Her main area of interest is genetics, and she is planning to pursue a master's degree in precision medicine.

## Supplementary Material

ytaf167_Supplementary_Data

## Data Availability

The data that support the findings of this case report are available from the corresponding author, upon reasonable request and subject to institutional review.

## References

[1] Medina de Chazal H, Del Buono MG, Keyser-Marcus L, Ma L, Moeller FG, Berrocal D, et al Stress cardiomyopathy diagnosis and treatment: JACC state-of-the-art review. J Am Coll Cardiol 2018;72:1955–1971. 30309474 10.1016/j.jacc.2018.07.072 PMC7058348

[2] Ghadri JR, Wittstein IS, Prasad A, Sharkey S, Dote K, Akashi YJ, et al International expert consensus document on Takotsubo syndrome (Part I): clinical characteristics, diagnostic criteria, and pathophysiology. Eur Heart J 2018;39:2032–2046. 29850871 10.1093/eurheartj/ehy076 PMC5991216

[3] Brinjikji W, El-Sayed AM, Salka S. In-hospital mortality among patients with Takotsubo cardiomyopathy: a study of the National Inpatient Sample 2008 to 2009. Am Heart J 2012;164:215–221. 22877807 10.1016/j.ahj.2012.04.010

[4] Pergolini A, Zampi G, Casali G, Madeo A, Visconti CL, Cipullo PL, et al Takotsubo syndrome after mitral valve replacement: case report and brief review of the literature. J Cardiothorac Vasc Anesth 2015;29:431–435. 24365714 10.1053/j.jvca.2013.09.005

[5] Kim YS, Lim JY. Risk factors for Takotsubo syndrome following cardiac surgery: a case-control study. J Card Surg 2021;36:2767–2773. 33993525 10.1111/jocs.15626

[6] Laghlam D, Touboul O, Herry M, Estagnasié P, Dib JC, Baccouche M, et al Takotsubo cardiomyopathy after cardiac surgery: a case-series and systematic review of literature. Front Cardiovasc Med 2023;9:1067444. 36704455 10.3389/fcvm.2022.1067444 PMC9871635

[7] Sendi P, Martinez P, Chegondi M, Totapally BR. Takotsubo cardiomyopathy in children. Cardiol Young 2020;30:1711–1715. 32843113 10.1017/S1047951120002632

